# Increased Skeletal Muscle Fat in Patients With Haematological Cancer Is Associated With Reduced Cardiorespiratory Fitness

**DOI:** 10.1002/jcsm.70186

**Published:** 2026-01-28

**Authors:** Nicholas J. Saner, Stephen J. Foulkes, Hayley T. Dillon, Lauren Burnham, Richard Thompson, Andre La Gerche, Mark J. Haykowsky, Erin J. Howden

**Affiliations:** ^1^ Baker Heart and Diabetes Institute Melbourne Victoria Australia; ^2^ Institute for Health and Sport Victoria University Melbourne Victoria Australia; ^3^ Heart, Exercise and Research Trials (HEART) Lab, St Vincent's Institute Fitzroy Victoria Australia; ^4^ iCARE Laboratory, College of Health Sciences, Faculty of Nursing University of Alberta Edmonton Alberta Canada; ^5^ Institute for Physical Activity and Nutrition, School of Exercise and Nutrition Sciences Deakin University Geelong Victoria Australia; ^6^ Department of Radiology and Diagnostic Imaging University of Alberta Edmonton Alberta Canada; ^7^ Cardiology Department St Vincent's Hospital Melbourne Fitzroy Victoria Australia; ^8^ Department of Cardiovascular Sciences KU Leuven Leuven Belgium; ^9^ HEART Lab Victor Chang Cardiovascular Research Institute Darlinghurst New South Wales Australia; ^10^ Department of Cardiometabolic Health University of Melbourne Melbourne Victoria Australia

**Keywords:** intermuscular fat, leukaemia, oxygen consumption, skeletal muscle

## Abstract

**Background:**

Patients with haematological cancer often exhibit reduced cardiorespiratory fitness and an elevated risk of cardiovascular disease. The mechanisms underlying this impairment are multifactorial, but the contribution of skeletal muscle fat infiltration has not been evaluated. This study aimed to compare thigh skeletal muscle fat fraction (SMFF) in patients with haematological cancer to healthy controls and to assess the contribution of SMFF to cardiorespiratory fitness in this cohort.

**Methods:**

We performed a cross‐sectional analysis of patients with haematological cancer (*n* = 70, 61% male, age: 51 ± 16 years) and age‐ and sex‐matched healthy controls (*n* = 70, 61% male, age: 50 ± 15 years). Thigh SMFF was assessed via magnetic resonance imaging. We also measured cardiorespiratory fitness (peak oxygen uptake, V̇O_2_peak), global longitudinal strain (GLS) via echocardiography and haemoglobin concentrations in the haematological cancer cohort. Hierarchical multiple regression analysis was performed to identify predictors of V̇O_2_peak.

**Results:**

SMFF was higher in the haematological cancer cohort versus the healthy control cohort (11.0% ± 3.4% vs. 8.8% ± 3.8%, *p* = 0.001). V̇O_2_peak was significantly lower than predicted values for the haematological cancer cohort (mean difference; 9.61 ± 8.30 mL^.^kg^−1.^min^−1^, *p* < 0.001). The multiple regression analysis accounted for 35% of the variance in V̇O_2_peak with both SMFF (*β* = −0.40, Δ*R* ^2^ = 0.14, *p* = 0.002) and haemoglobin concentrations (*β* = 0.50, Δ*R* ^2^ = 0.23, *p* < 0.001) being significant independent predictors of V̇O_2_peak, while skeletal muscle volume (*β* = 0.00, Δ*R* ^2^ = 0.00, *p* = 0.767), GLS (*β* = 0.06, Δ*R* ^2^ = 0.00, *p* = 0.509), prior anthracycline treatment (*β* = 0.00, Δ*R* ^2^ = 0.00, *p* = 0.962) and clinical diagnosis (*β* = 0.00, Δ*R* ^2^ = 0.00, *p* = 0.555) were not.

**Conclusions:**

SMFF is increased in haematological cancer patients and contributes to reduced V̇O_2_peak. Consequently, increased SMFF may be an important target to improve cardiovascular health and cardiorespiratory fitness in this population.

## 1 Introduction

Recent improvements in the treatment of haematological cancer have led to increased rates of survival [1], despite this, these survivors have reduced peak aerobic power, measured objectively as decreased peak oxygen uptake (V̇O_2_peak), via cardiopulmonary exercise testing [2, 3, 4]. V̇O_2_peak is an integrative measure of cardiovascular, pulmonary, haematologic, and skeletal muscle function, and is an independent predictor of all‐cause and cardiovascular mortality in healthy and clinical populations, including cancer patients [5, 6, 7]. Although the mechanisms underpinning reduced V̇O_2_peak (defined as < 85% of age predicted values) are not well understood, impairments in cardiovascular and skeletal muscle function associated with anti‐cancer treatments and sedentary deconditioning have been implicated [8, 9]. Improving our understanding of these mechanisms will help to better inform therapeutic approaches capable of improving patient outcomes in this context.

Traditionally, studies examining the mechanisms of reduced V̇O_2_peak in cancer survivors have predominantly focused on central (cardiac) limitations [9, 10, 11]. However, recent research in haematological [3, 12, 13, 14] and breast cancer patients [8, 10, 15] suggests that skeletal muscle factors, and specifically, excessive muscle fat infiltration (myosteatosis) are likely important and underappreciated determinants of the reduced V̇O_2_peak. The aggressive treatment approaches for haematological cancer, combined with prolonged hospitalisation with periods of extended bed rest and disease‐specific effects, increase the risk of myosteatosis in this population [16, 17]. Indeed, skeletal muscle fat fraction (SMFF) is reported to increase following anthracycline‐based chemotherapy [18], a common treatment for patients with haematological cancer. However, whether SMFF is increased compared with healthy populations or is a determinant of V̇O_2_peak in patients with haematological cancer has not been established.

Accordingly, we performed a cross‐sectional analysis on a cohort of adult patients with haematological cancer, primarily consisting of acute myeloid or lymphoblastic leukaemia and myelodysplasia, many of whom had recently undergone treatment with anti‐cancer therapies. Our study aimed to examine whether SMFF is increased in patients with haematological cancer compared with age‐, sex‐ and body mass index (BMI)‐matched healthy controls and if SMFF contributes to reduced V̇O_2_peak in this cohort. We hypothesised that haematological cancer patients have increased SMFF and that this significantly contributes to the reduced whole‐body V̇O_2_peak, independently of central determinants of cardiorespiratory fitness.

## 2 Methods

### 2.1 Participants

This cross‐sectional study consisted of 140 adults (≥ 18 years), including 70 individuals with a haematological cancer undergoing treatment at the Alfred Hospital (Melbourne, Australia) that had been invited to participate in the Allo‐Active (ACTRN: 12619000741189) or IMPROVE (ACTRN: 12623001049662) clinical trials and 70 age‐, sex‐ and BMI‐matched healthy control participants recruited from the community at the University of Alberta. Exclusion criteria for the haematological cancer cohort included contraindications for magnetic resonance imaging (MRI) and inability to communicate or read English. Healthy adults without contraindications to MRI were recruited from the local community and considered for inclusion in the healthy control group, with exclusion for a history of cancer, cardiovascular or lung disease, hypertension, metabolic syndrome, BMI ≥ 40 kg/m^2^ or conditions requiring regular medication use for a chronic condition. The experimental procedures were explained to all participants, with written informed consent provided as approved by the Alfred Human Research Ethics Committee or the University of Alberta Health Research Ethics Board. Baseline characteristics of participants are presented in Table 1.

**TABLE 1:** Participant characteristics.

Characteristics	Haematological cancer (*n* = 70)	Healthy control (*n* = 70)	*p*
Age (years)	51 ± 16	50 ± 15	0.788
Males (%)	43 (61)	43 (61)	0.569
Height (cm)	171.8 ± 8.9	171.2 ± 9.8	0.715
Weight (kg)	80.6 ± 17.8	79.3 ± 11.5	0.592
BMI (kg^.^m^2^)	27.3 ± 5.8	27.0 ± 3.2	0.742
6MWT (m)	582 ± 80	619 ± 88	**0.009**
Cardiovascular risk factors, *n* (%)			
Overweight or obese	42 (60)	45 (64)	0.601
Hypertension	10 (14)	0 (0)	
Hyperlipidaemia/dyslipidaemia	6 (8)	0 (0)	
Type 2 diabetes	5 (7)	0 (0)	
Prior CVD event/diagnosis	8 (11)	0 (0)	
Hypothyroidism	3 (4)	0 (0)	
> 1 risk factor	27 (38)	0 (0)	
Haematological cancer diagnosis, *n* (%)		N/A	
Acute myeloid leukaemia	27 (38)		
Acute lymphocytic leukaemia	12 (17)		
Chronic myeloid leukaemia	6 (9)		
Chronic lymphocytic leukaemia	1 (1)		
Myelodysplasia	13 (19)		
Myeloproliferative disorder	3 (4)		
Hodgkin's lymphoma	2 (3)		
Other leukaemias	6 (9)		
Prior cancer treatment, *n* (%)		N/A	
No prior cancer treatment	5 (7)		
Chemotherapy	60 (85)		
Prior anthracyclines	42 (60)		
Targeted therapy	26 (37)		
Immunotherapy	14 (20)		
Alkylating agents	15 (21)		
Antimetabolites	50 (71)		
Vinca alkyloids	14 (20)		
Other antineoplastics	5 (7)		

*Note:* Data represent mean ± SD or *n* (%). *p* < 0.05 denotes statistical significance.

Abbreviations: 6MWT, 6‐min walk test; BMI, body mass index; CVD, cardiovascular disease; N/A, not applicable.

### 2.2 Anthropometric Measures

Height (cm) and body mass (kg) were assessed using a fixed stadiometer (SECA, Hamburg, Germany) and portable scales (Coverall Medical Technologies, Victoria, Australia), respectively, and used to calculate BMI (kg^.^m^−2^).

### 2.3 Cardiopulmonary Exercise Testing

Participants from the haematological cancer cohort performed a maximal cardiopulmonary exercise test (CPET) with expired gas analysis (Vyntus CPX, Carefusion, USA) on an electronically braked cycle ergometer (Lode Excalibur Sport, Groningen, the Netherlands) for the measurement of V̇O_2_peak [19]. Following ~3–5 min of unloaded cycling, an incremental ramp protocol was conducted (5–25 W/min), which was individualised to the participant's age, weight and self‐reported physical activity levels. Standard criteria were applied for assessment of achieving V̇O_2_peak (i.e., meeting two of the following criteria: volitional fatigue, respiratory exchange ratio [RER] > 1.1, > 85% age predicted heart rate maximum and/or blood lactate concentration > 8 L^−1^). V̇O_2_peak was defined as the average of the six consecutive highest 5 s V̇O_2_ values (i.e., the V̇O_2_peak from a rolling 30 s average). Participants were categorised as having reduced cardiorespiratory fitness if their V̇O_2_peak was < 85% of the age‐, sex‐ and weight‐predicted value derived from the FRIEND registry reference equation [20]. Functional disability was defined as V̇O_2_peak < 18 mL^.^kg^−1.^min^−1^ as per the American Heart Association Scientific Statement [21, 22, 23].

### 2.4 Aerobic Endurance

The 6‐min walk test (6MWT) was performed in accordance with American Thoracic Guidelines in both the haematological cancer and healthy control cohorts.

### 2.5 Skeletal Muscle Thigh MRI

**FIGURE 1:**
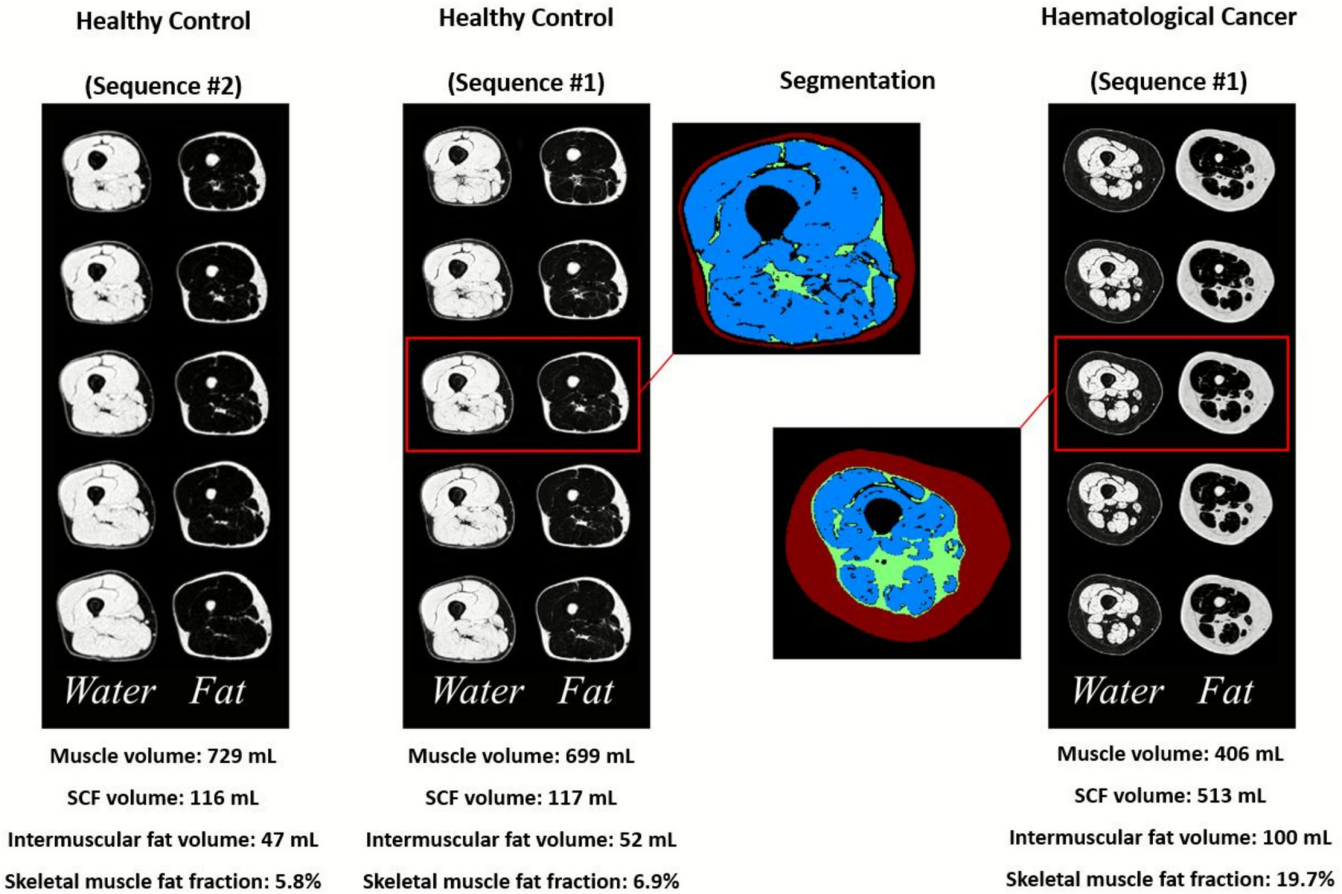
Illustrative fat‐ and water‐separated images in a healthy control and haematological cancer patient. Acquisitions were repeated in the same control subject using the two different pulse sequence variations used in the two study cohorts. Muscle and fat volumes and fat fractions calculated using an automated machine learning segmentation approach are reported for each case. The five transverse slices cover 59.5 mm, centred 17.5 cm from the distal head of the femur. Abbreviation: SCF, subcutaneous fat.

MRI scans (3T MAGNETOM Prisma; Siemens Healthcare; Erlangen, Germany) of the right thigh were acquired in the supine position, from the superior patella to the greater trochanter using the two‐point Dixon method to reconstruct fat and water separated images. Scans used a transverse slice orientation with 0.75‐mm in‐plane resolution and 5‐mm slice thickness. A similar fat‐ and water‐separated acquisition (also on a 3T MAGNETOM Prisma) was used in the control cohort with 1.0‐mm in‐plane resolution and 3.5‐mm slice thickness. A subset of images with a standardised spatial coverage, including 59.5‐mm centred at 17.5 cm from the distal head of the femur, was used for all cancer patient and control analyses (Figure 1).

For image segmentation, a custom machine learning approach was used to perform automated analysis to identify regions of subcutaneous fat, thigh skeletal muscle, intermuscular fat and bones (Figure 1). Volumes for each component were quantified using the disk summation method. The skeletal muscle and intermuscular fat volumes were used to calculate the muscle fat fraction as the ratio of fat to the total muscle and intermuscular fat volume [intermuscular fat/(intermuscular fat + muscle) × 100%].

### 2.6 Resting Echocardiography

Resting cardiac function was evaluated via echocardiography (Vivid E95, GE HealthCare, Horten, Norway) in the haematological cancer cohort. Images were collected, saved in a digital format and analysed offline (Echopac v13.0.00, GE, Norway) by a trained sonographer. A three‐dimensional full‐volume dataset was acquired to measure left ventricular ejection fraction (LVEF). Two‐dimensional speckle tracking echocardiography‐derived global longitudinal strain (GLS) was quantified from three apical views at a temporal resolution of 60–90 frames per second with GLS defined as the average negative value of the strain rate curves.

### 2.7 Blood Analysis

Fasting blood samples were collected to assess haemoglobin concentrations in the haematological cancer group. Additional markers of myocardial injury (Troponin‐1; cTn‐I) and stress (B‐natriuretic peptide; BNP) were also assessed in the haematological cancer group at the Alfred Health NATA‐accredited pathology laboratory.

### 2.8 Statistical Analysis

Characteristics between the haematological cancer group and healthy control group were compared using independent *t*‐tests, with Bonferroni correction for multiple comparisons. In the case of nonparametric distribution of continuous variables (confirmed by the Shapiro–Wilk test), the Mann–Whitney *U* test was used. Chi‐square tests were performed to assess differences between categorical variables. The associations between SMFF and aerobic endurance in both the haematological cancer group and the healthy control group were assessed by linear regression analysis. Linear regression was also used to assess the relationship between V̇O_2_peak and SMFF, haemoglobin, LVEF and GLS in the haematological cancer group. Hierarchical multiple regression analysis was used to determine the contribution of demographic (age and sex), physiological (skeletal muscle volume, SMFF, haemoglobin and GLS) and clinical (prior anthracycline exposure and clinical diagnosis) factors to the V̇O_2_peak (mL kg^−1^ min^−1^) observed in the haematological cancer group. Sensitivity analysis was performed by replacing muscle volume with BMI and by replacing SMFF with intermuscular fat volume. All data are presented as mean ± SD, with *p* ≤ 0.05 indicating statistical significance. Statistical analyses were conducted using IBM SPSS statistics version 28.0.1.0 (IBM Corp. Armonk, NY, USA). Figures were created using Prism, version 10.2.3 (GraphPad Software Inc., San Diego, CA, USA).

## 3 Results

### 3.1 Participant Characteristics

There were no significant differences in baseline anthropometric characteristics between groups (Table 1). Sixty‐five (93%) individuals from the haematological cancer cohort had received prior anti‐cancer treatment, 60 (85%) of whom had received prior chemotherapy, of those, 42 (60%) had received anthracycline‐based chemotherapy (median anthracycline dose equivalent = 180 mg/m^2^, [range 100–380 mg/m^2^], median time since anthracycline completion = 124 days, [range 32–1064 days]). Forty‐two patients in the haematological cancer group and 45 participants from the healthy control group were classified as being overweight or obese (BMI > 25 kg m^−2^, *p* = 0.601). Twenty‐seven (39%) of the haematological cancer cohort had > 1 traditional cardiovascular disease risk factor.

### 3.2 Skeletal Muscle Composition

There were several differences in MRI‐derived thigh skeletal muscle composition between the haematological cancer cohort and the healthy control cohort (Table 2). Thigh SMFF and intermuscular fat volume were higher in the haematological cancer cohort compared with the healthy control cohort. There were no differences in subcutaneous fat or muscle volume between cohorts.

**TABLE 2:** Thigh skeletal muscle composition analysis in patients with haematological cancer and healthy controls.

	Haematological cancer (*n* = 70)	Healthy control (*n* = 70)	Mean difference [95% CI]	*p*
Subcutaneous fat (mL)	355 ± 216	299 ± 201	56 ± 294 [−13, 125]	0.113
Muscle volume (mL)	628 ± 229	624 ± 118	4 ± 257 [−57, 65]	0.898
IMF volume (mL)	79 ± 39	58 ± 20	20 ± 43 [10, 31]	**< 0.001**
SMFF (%)	11.0 ± 3.4	8.8 ± 3.8	2.2 ± 5.4 [0.9, 3.5]	**0.001**

*Note:* Data represent mean ± SD, *p* < 0.05 denotes statistical significance.

Abbreviations: IMF, intermuscular fat; SMFF, skeletal muscle fat fraction.

**FIGURE 2:**
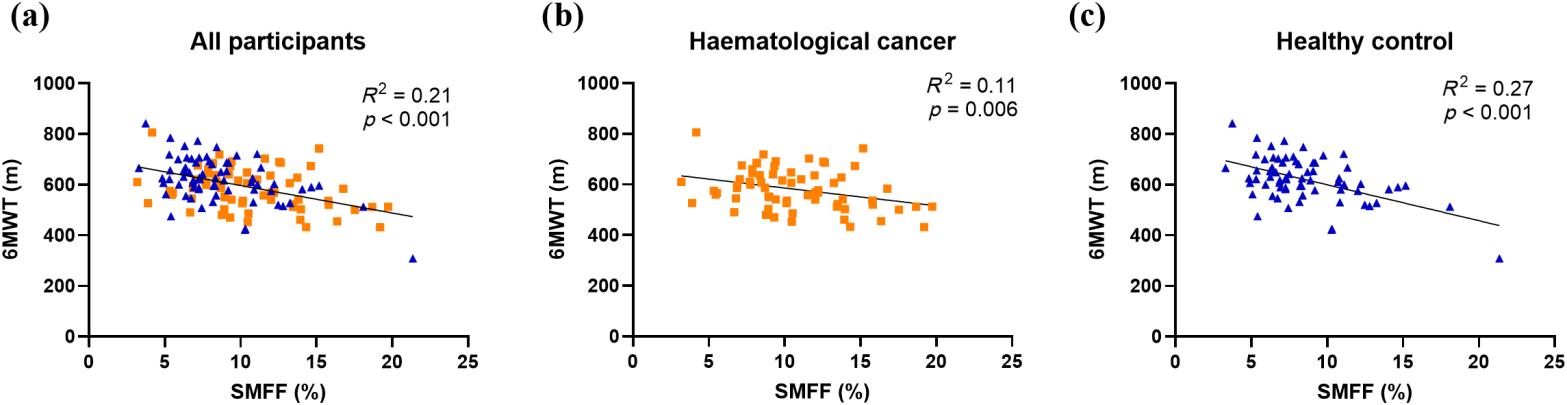
The relationship between skeletal muscle fat fraction (SMFF, %) and six‐minute walk test (6MWT) distance in (a) all participants, (b) the haematological cancer cohort (■) and (c) healthy control cohort (▲).

The 6MWT distance was lower in the haematological cancer cohort (582 ± 80 m) compared with the healthy control group (619 ± 88 m) (mean difference ± SD, [95% CI], −38 ± 119 m, [−67 to −9 m], *p* = 0.009). Linear regression revealed that SMFF was inversely correlated with 6MWT distance (Figure 2).

### 3.3 Cardiorespiratory Fitness in the Haematological Cancer Cohort

**FIGURE 3:**
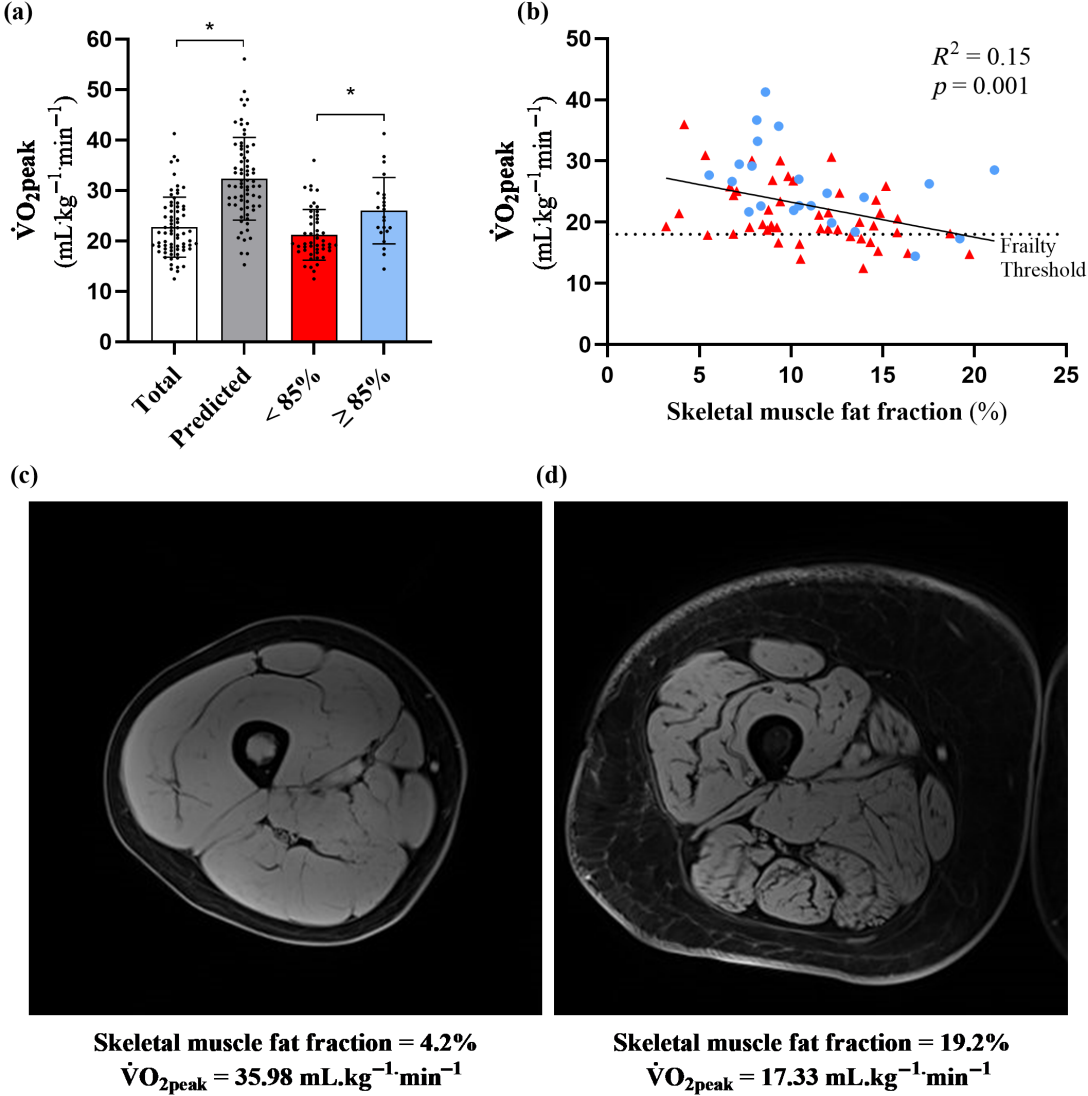
The relationship between peak oxygen uptake (V̇O_2_peak) and skeletal muscle fat fraction in a haematological cancer cohort. (a) V̇O_2_peak of the haematological cancer cohort (total; *n* = 70) compared with the age‐, sex‐ and weight‐predicted V̇O_2_peak, and separated into reduced (< 85%, *n* = 48) and normal (> 85%, *n* = 22) predicted V̇O_2_peak. (b) Linear regression between V̇O_2_peak and thigh skeletal muscle fat fraction (%); ▲, < 85% predicted V̇O_2_peak; ●, ≥ 85% predicted V̇O_2_peak. (c) Representative image of a participant with (c) low and (d) high thigh skeletal muscle fat fraction and their respective V̇O_2_peak. *Represents statistically significant difference between total and predicted V̇O_2_peak values, and between < 85% predicted and ≥ 85% predicted cohorts, *p* < 0.05.

The haematological cancer cohort achieved a mean V̇O_2_peak of 22.72 ± 5.94 mL^.^kg^−1.^min^−1^, which was 30% lower than predicted (32.32 ± 8.22 mL^.^kg^−1^^.^min^−1^) for the cohort (mean difference; 9.61 ± 8.30 mL^.^kg^−1^^.^min^−1^ [7.62 to 11.58 mL^.^kg^−1.^min^−1^], *p* < 0.001) (Table 3). The majority of participants (*n* = 48: 69%) were considered to have reduced V̇O_2_peak (< 85% predicted V̇O_2_peak), with 13 participants (19%) falling below the threshold for functional disability (V̇O_2_peak < 18^.^mL kg^−1.^min^−1^) (Figure 3) [23]. Participants with < 85% predicted V̇O_2_peak were also younger, achieved a lower peak power output and had a lower BMI and haemoglobin levels, compared with participants who achieved > 85% predicted V̇O_2_peak (Table 3). However, SMFF, skeletal muscle volume and total body weight were similar.

**TABLE 3:** Haematological cancer cohort with reduced (< 85% predicted) and normal (≥ 85% predicted) V̇O_2_peak.

	Total (*n* = 70)	< 85% V̇O_2_peak (*n* = 48)	≥ 85% V̇O_2_peak (*n* = 22)	*p*
Participant characteristics				
Age (years)	50 ± 16	48 ± 17	57 ± 14*	**0.017**
Males (%)	43 (61)	32 (66)	11 (50)	0.199
Height (cm)	171.8 ± 8.9	172.4 ± 8.6	170.4 ± 9.8	0.392
Weight (kg)	80.6 ± 17.8	78.3 ± 16.3	85.7 ± 20.4	0.110
BMI (kg^.^m^−2^)	27.3 ± 5.8	26.3 ± 5.0	29.5 ± 6.9*	**0.031**
Cardiorespiratory fitness				
V̇O_2_peak (mL^.^kg^−1.^min^−1^)	22.72 ± 5.94	21.21 ± 5.02	26.43 ± 6.27*	**0.001**
V̇O_2_peak (L^.^min^−1^)	1.80 ± 0.55	1.64 ± 0.47	2.19 ± 0.56*	**< 0.001**
Peak power (W)	157.6 ± 54.2	146.2 ± 45.33	185.9 ± 62.85*	**0.008**
Peak heart rate (bpm)	169 ± 20	168 ± 22	172 ± 15	0.396
RER	1.42 ± 0.15	1.45 ± 0.15	1.38 ± 0.11	0.110
Functional disability, *n* (%)	13 (19)	11 (23)	2 (9)	0.167
6MW distance (m)	584.9 ± 80.1	573.6 ± 84.9	608.6 ± 64.7	0.108
MRI skeletal muscle analysis				
Subcutaneous fat (mL)	355 ± 216	330 ± 194	410 ± 253	0.147
Skeletal muscle volume (mL)	628 ± 229	623 ± 260	639 ± 143	0.785
Intermuscular fat volume (mL)	79 ± 39	78 ± 42	80 ± 33	0.877
Skeletal muscle fat fraction (%)	11.0 ± 3.4	10.9 ± 3.8	11.2 ± 4.2	0.787
Resting cardiac function				
LVEF (%)	58.9 ± 6.0	59.0 ± 6.2	58.5 ± 5.7	0.747
GLS (%)	−18.6 ± 2.6	−18.4 ± 2.6	−19.17 ± 2.4	0.258
Blood analysis				
Haemoglobin (g^.^L^−1^)	115 ± 21	111 ± 21	125 ± 18*	**0.007**
Troponin I (ng^.^L^−1^)	5 ± 10	6 ± 12	4 ± 6	0.457
BNP (ng^.^L^−1^)	34 ± 59	41 ± 72	19 ± 10	0.174

Abbreviations: BNP, B‐natriuretic peptide; GLS, global longitudinal strain; LVEF, left ventricular ejection fraction; RER, respiratory exchange ratio; V̇O_2_peak, peak oxygen consumption, functional disability—< 18 mL^.^kg^−1.^min^−1^.

* Significantly different from < 85% predicted V̇O_2_peak group, *p* < 0.05.

### 3.4 Impact of Anthracycline Treatment on V̇O_2_peak, SMFF, Haemoglobin and GLS

In patients with (*n* = 42) or without (*n* = 28) prior anthracycline (AC) treatment there were no significant differences in V̇O_2_peak (no AC, 23.02 ± 6.31 mL^.^kg^−1.^min^−1^; AC, 22.55 ± 5.79 mL^.^kg^−1.^min^−1^ [−3.44 to 2.51 mL^.^kg^−1.^min^−1^], *p* = 0.755), SMFF (no AC, 11% ± 3%; AC, 11% ± 4% [−2% to 1%], *p* = 0.727), haemoglobin (no AC, 113.1 ± 22.2 g^.^L^−1^; AC, 116.7 ± 19.6 g^.^L^−1^ [−6.7 to 13.8 g^.^L^−^1], *p* = 0.492) or GLS (no AC, −19.0% ± 2.8%; AC, −18.3% ± 2.4% [−0.6% to 2.0%], *p* = 0.287).

### 3.5 Predictors of V̇O_2_peak in the Haematological Cancer Cohort

**FIGURE 4:**
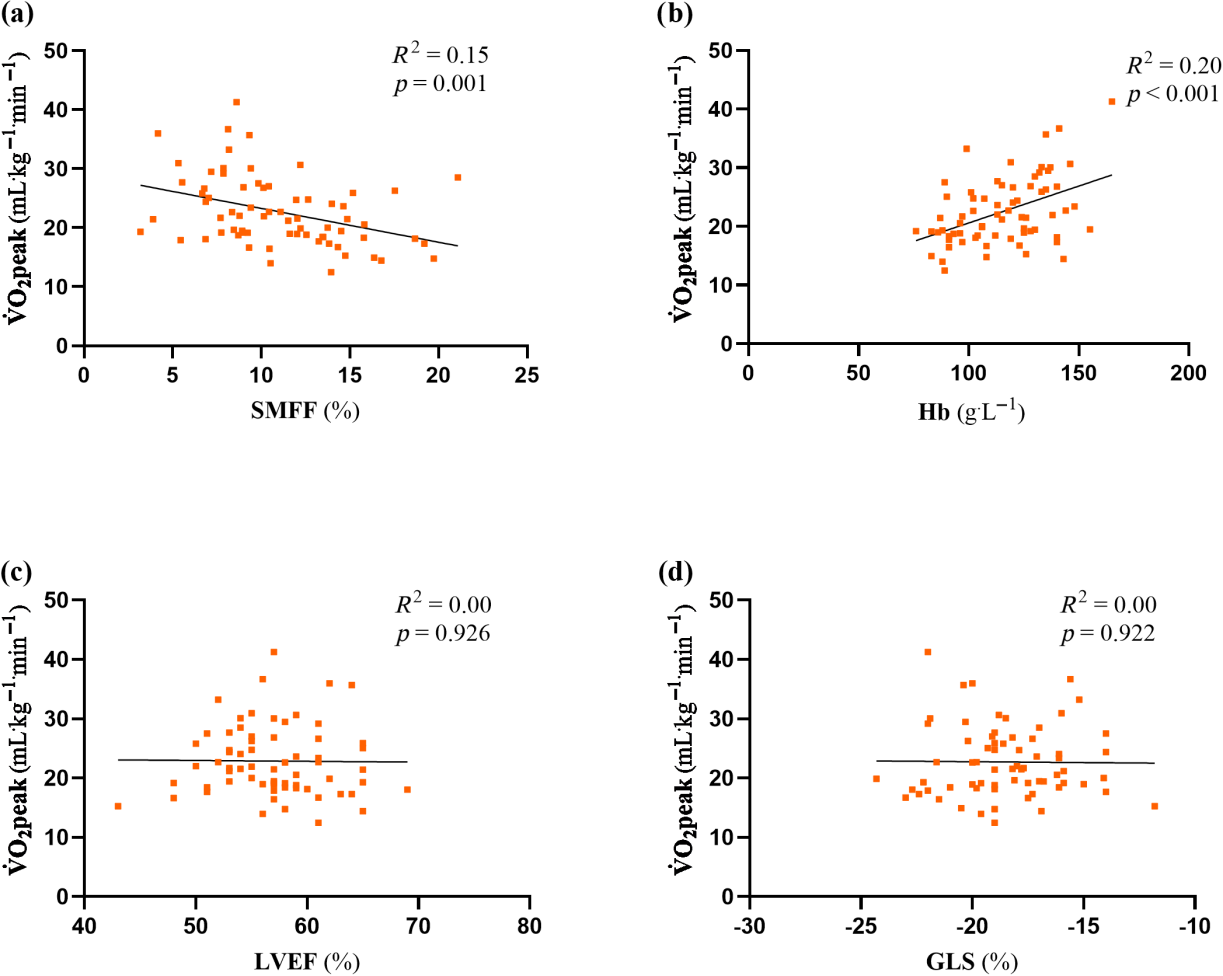
Predictors of V̇O_2_peak—simple linear regression analysis comparing V̇O_2_peak (mL^.^kg^−1.^min^−1^) with (a) skeletal muscle fat fraction (SMFF; %), (b) haemoglobin (Hb;g^.^L), (c) left ventricular ejection fraction (LVEF; %) and (d) global longitudinal strain (GLS; %), in the haematological cancer cohort. *R* ^2^, adjusted coefficient of determination.

SMFF was inversely associated with V̇O_2_peak (*R* ^2^ = 0.15, *p* = 0.001) in the haematological cancer cohort (Figure 4). Blood haemoglobin concentrations were positively associated with V̇O_2_peak (*R* ^2^ = 0.25, *p* < 0.001). However, measures of resting left ventricular systolic function (LVEF, *R* ^2^ = 0.00, *p* = 0.913; GLS, *R* ^2^ = 0.00, *p* = 0.898) were not. To assess the contribution of both central and peripheral determinants of V̇O_2_peak (mL^.^kg^−1.^min^−1^) within the haematological cancer cohort a hierarchical multiple linear regression analysis was created (Table 4), with the models including age, sex, muscle volume, SMFF, haemoglobin, GLS, prior anthracycline treatment, and haematological cancer diagnosis as predictors. These variables accounted for 35% of variance in the model. Overall, the addition of SMFF and haemoglobin to the model accounted for 14% and 23% of variance in the model, respectively; and both were independently associated with changes in V̇O_2_peak (SMFF, *β* = −0.37, *p* < 0.002; haemoglobin, *β* = 0.49, *p* < 0.001). However, age (*β* = −0.13, *p* = 0.268), sex (*β* = −0.21, *p* = 0.0.096), skeletal muscle volume (*β* = 0.04, *p* = 0.767), GLS (*β* = 0.00, *p* = 0.973), prior anthracycline treatment (*β* = 0.01, *p* = 0.962), and clinical diagnosis (*β* = −0.07, *p* = 0.555) were not associated with changes in V̇O_2_peak. We performed a sensitivity analysis of the model by replacing muscle volume with BMI, however, SMFF remained a significant independent predictor of V̇O_2_peak (*β* = −0.32, Δ*R* ^2^ = 0.07, *p* = 0.024). In addition to this, we included intermuscular fat volume in the model, instead of SMFF, with intermuscular fat volume also being a significant predictor of V̇O_2_peak (*β* = −0.51, Δ*R* ^2^ = 0.16, *p* < 0.001).

**TABLE 4:** Hierarchical multiple regression analysis summary predicting changes in V̇O_2_peak (mL^.^kg^−1.^min^−1^) with age, sex, muscle volume, SMFF, haemoglobin, GLS, prior anthracycline treatment and clinical diagnosis.

Model	Unstandardised *β*	SE of *β*	Standardised *β*	*p*	Adjusted *R* ^2^	Δ Adjusted *R* ^2^
Age (years)	−0.1	0.0	−0.1	0.268	0.00	0.02
Age (years)	−0.1	0.1	−0.2	0.096	0.03	0.04
Sex (m/f)	2.5	1.5	0.2			
Age (years)	−0.1	0.1	−0.2	0.767	0.02	0.00
Sex (m/f)	2.3	1.6	0.2			
Muscle volume (mL)	0.0	0.0	0.0			
Age (years)	−0.1	0.0	−0.2	**0.002**	0.15	0.14
Sex (m/f)	2.2	1.5	0.0			
Muscle volume (mL)	0.0	0.0	0.0			
SMFF (%)	−0.6	0.2	−0.4			
Age (years)	−0.1	0.0	−0.2	**< 0.001**	0.38	0.23
Sex (m/f)	2.4	1.3	0.2			
Muscle volume (mL)	−0.0	0.0	−0.1			
SMFF (%)	−0.6	0.1	−0.4			
Haemoglobin (g^.^L^−1^)	0.1	0.0	0.5			
Age (years)	−0.1	0.0	−0.2	0.945	0.37	0.00
Sex (m/f)	2.4	1.3	0.2			
Muscle volume (mL)	−0.0	0.0	−0.1			
SMFF (%)	−0.6	0.2	−0.4			
Haemoglobin (g^.^L^−1^)	0.1	0.0	0.5			
GLS (%)	0.0	0.3	0.0			
Age (years)	−0.1	0.0	−0.2	0.962	0.36	0.00
Sex (m/f)	2.5	1.5	0.2			
Muscle volume (mL)	−0.0	0.0	−0.1			
SMFF (%)	−0.6	0.2	−0.4			
Haemoglobin (g^.^L^−1^)	0.1	0.0	0.5			
GLS (%)	0.0	0.3	0.0			
Anthracyclines (Y/N)	0.1	1.4	0.0			
Age (years)	−0.1	0.0	−0.2	0.555	0.35	0.00
Sex (m/f)	2.6	1.5	0.2			
Muscle volume (mL)	−0.0	0.0	−0.1			
SMFF (%)	−0.6	0.2	−0.4			
Haemoglobin (g^.^L^−1^)	0.1	0.0	0.5			
GLS (%)	0.0	0.3	0.0			
Anthracyclines (Y/N)	−0.2	1.4	−0.0			
Clinical diagnosis	−0.2	0.3	−0.0			

Abbreviations: f, female; GLS, global longitudinal strain; m, male; SMFF, skeletal muscle fat fraction.

## 4 Discussion

The major new finding in this study is that SMFF is significantly higher in patients with haematological cancer compared with age‐, sex‐ and BMI‐matched healthy control participants. Additionally, predicted V̇O_2_peak was reduced in the haematological cancer patients and both SMFF and haemoglobin were independent predictors of V̇O_2_peak in this cohort, while resting measures of left‐ventricular systolic function were not. Given the prognostic link between V̇O_2_peak and cardiovascular morbidity and mortality, these findings provide insight regarding targets for therapeutic interventions capable of improving patient outcomes in survivors of haematological cancer.

To our knowledge, this is the first study to demonstrate that haematological cancer patients exhibit increased SMFF, which is inversely associated with aerobic endurance (in both cancer and healthy control groups) and V̇O_2_peak in cancer survivors. Although these are novel data for this population, several other studies have reported similar observations of increased muscle fat infiltration in breast cancer survivors [8, 10, 15, 18] and people with heart failure [24, 25]; and increased SMFF has been shown to be a strong predictor of V̇O_2_peak in these cohorts. For example, Reding et al. [8] reported that a small mixed cohort of breast cancer (*n* = 8) and lymphoma (*n* = 6) survivors had increased paraspinal muscle fat infiltration, which was an independent predictor of reduced V̇O_2_peak. Beaudry et al. [15] also showed a strong relationship between calf, thigh and paraspinal fat content and V̇O_2_peak in a cohort of breast cancer survivors who had received anthracycline chemotherapy. Therefore, our findings align with these previous reports and extend this evidence to patients with haematological cancer, many of whom have recently undergone anti‐cancer therapies. These findings suggest that SMFF could be used as an imaging biomarker with prognostic utility, identifying patients at increased risk of functional limitation and cardiovascular disease.

The increased SMFF in this cohort is likely caused by several factors, stemming from the interplay of anti‐cancer therapies, disease‐related metabolic disruptions and associated sedentary deconditioning. Chemotherapy‐induced increases in SMFF have previously been reported [18] and have been associated with impaired oxygen extraction in lower leg muscles during exercise [15]. Our findings complement previous research that has demonstrated reduced arteriovenous oxygen differences in patients undergoing allogeneic stem cell transplant for their haematological cancer [3]. Increases in SMFF have been hypothesised to contribute to reduced V̇O_2_peak by impairing oxygen diffusion capacity in the muscle and limiting the supply of oxygen to the mitochondria [9, 26, 27]. Additionally, increases in SMFF may also be the result of impaired mitochondrial fatty acid oxidation [28]. Indeed, reductions in markers of skeletal muscle mitochondrial content [29] and oxidative phosphorylation (via ^31^P magnetic resonance spectroscopy) [30] in patients following chemotherapy treatment for breast cancer have been reported previously. Taken together, this may suggest that chemotherapy‐induced reductions in mitochondrial oxidative capacity underpin increased SMFF and reduced V̇O_2_peak observed in this cohort. Collectively, our findings provide important insight regarding the potential mechanisms of reduced V̇O_2_peak and which likely contributes to the increased incidence of cardiovascular and metabolic disease in this patient group. However, further research to establish a causative role of SMFF for reduced oxygen extraction and direct measures of oxygen utilisation (e.g., mitochondrial respiratory function in permeabilised muscle fibres) are required.

Reduced functional capacity (V̇O_2_peak) has been observed in patients with haematological cancers previously [2, 3, 4, 14, 31]. Our current data suggest that these reductions in V̇O_2_peak may persist beyond treatment (time since treatment cessation ~4 months). Indeed, within this current cohort, 69% of patients were classified as having a reduced V̇O_2_peak compared with predicted values (V̇O_2_peak was 70% of the predicted values given by the FRIEND reference equation [20]), which is known to be an independent predictor of all‐cause mortality in cancer survivors [32]. Dillon et al. [3] previously reported that patients scheduled for allogeneic stem cell transplant (*n* = 17) also had markedly reduced V̇O_2_peak (mean V̇O_2_peak = 22.9 mL.kg^−1.^min^−1^, 67% of the age‐predicted value). Furthermore, 19% of the current cohort had a V̇O_2_peak below the threshold for functional disability, which is associated with a 7‐to‐9‐fold increased risk of cardiovascular disease mortality [2, 22, 33]. This threshold is associated with a reduced ability to perform tasks independently and suggests that even basic activities of daily living require near maximal physical efforts [21]. These findings highlight the importance of understanding the factors that underpin reduced V̇O_2_peak in these cohorts so that targeted approaches to improve cardiorespiratory fitness and patient outcomes can be devised. Furthermore, the optimal timing of interventions needs to be determined given that our data suggest the impairment of V̇O_2_peak persists after cessation of treatment.

The multiple regression analysis indicated that haemoglobin concentrations are a significant predictor of V̇O_2_peak in the haematological cancer cohort. Haemoglobin concentrations were lower in patients classified as having reduced V̇O_2_peak in this study. Anaemia is common in patients with haematological cancer and may result from the disease itself, or the associated treatments [34]. Decreased haemoglobin levels have been associated with increased fatigue [35], which may culminate in increased sedentary behaviour and exacerbate increases in SMFF and reductions in V̇O_2_peak [17]. Our data also indicate that there was no association between resting GLS and V̇O_2_peak in this cohort of haematological cancer patients. This is in contrast to a previous study in haematological cancer survivors that underwent allogeneic stem cell transplantation, which reported that LVEF (an additional measure of left ventricular systolic function) was significantly associated with predicted V̇O_2_peak [36]. While assessments of resting cardiac function are the current standard of care for this cohort, our group has previously demonstrated that resting echocardiographic assessments do not detect reductions in cardiac reserve, which can be detected by utilising more sensitive exercise cardiac MRI, in patients undergoing allogeneic stem cell transplant [3, 4, 14]. Therefore, our findings that resting measures of left ventricular systolic function do not predict V̇O_2_peak are in line with previous research, but highlight the advantages of implementing exercise‐based cardiac assessments.

Future research that investigates the potential of targeted interventions for improving central and peripheral determinants of V̇O_2_peak in this cohort is essential. Recently, studies in both breast cancer and allogeneic stem cell transplantation patients have demonstrated that personalised exercise training interventions (involving both endurance and resistance exercise) can ameliorate the deleterious effects of anti‐cancer treatments on V̇O_2_peak and cardiac reserve [4, 37, 38]. Whether these longitudinal improvements in V̇O_2_peak are associated with changes in SMFF remains to be determined. Our results suggest that longitudinal assessments of SMFF may provide insight regarding the efficacy of treatment approaches, particularly with exercise‐based interventions.

### 4.1 Study Limitations

Due to the cross‐sectional nature of the study, we are unable to directly link increases in SMFF with disease progression or the anti‐cancer therapies received by the participants. A longitudinal approach is also required to link changes in V̇O_2_peak with changes in SMFF. Similarly, our suggestion that V̇O_2_peak does not return to predicted levels after treatment requires qualification given the cross‐sectional nature of the observations. To provide additional insight regarding the central determinants of V̇O_2_peak in this cohort, exercise‐based measures of cardiac function, such as exercise‐cardiac MRI, should be included in future studies. Although there were slight cross‐site differences in MRI protocols, previous work comparing different sequences for the measurement of intramuscular fat and muscle volumes suggests biases of ~1 mL or less and strong agreement between methods (*R* ^2^ = 0.90–0.99), supporting the robustness of this approach [39]. The imaging methods used in this study are not sensitive to the intramuscular fat pool (i.e., interstitial fat and deposits too small to see with MRI), which may be substantial. Future studies may also consider including measures of habitual physical activity, which may contribute to the findings.

## 5 Conclusions

Collectively, our findings suggest that SMFF is increased in patients with haematological cancers, many of whom have recently undergone anti‐cancer treatment and that SMFF may be a contributing and underappreciated mechanism linking reduced V̇O_2_peak to heart failure and premature mortality in this cohort. These data highlight the need for further research into therapeutic interventions that can target skeletal muscle fat and ameliorate impairments in V̇O_2_peak in haematologic cancer survivors.

## Funding

This work was supported by a project grant from the World Cancer Research Fund (Reference #2019_1666). E.J.H. is supported by an Australian National Heart Foundation Future Leader Fellowship (ID: 102536). A.L.G. is supported by an NHMRC Investigator Grant (APP2027105).

## Conflicts of Interest

The authors declare no conflicts of interest.

## Data Availability

De‐identified data and analysis code are available on Zenodo at (DOI: 10.5281/zenodo.17189532), with accompanying documentation to support reproducibility.
